# Treatment and Outcomes of Infections Caused by Diverse Carbapenemase-Producing Carbapenem-Resistant *Enterobacterales*

**DOI:** 10.3389/fcimb.2020.579462

**Published:** 2020-10-14

**Authors:** Fang Kang Lim, Yi Xin Liew, Yiying Cai, Winnie Lee, Jocelyn Q. M. Teo, Wei Qi Lay, Jasmine Chung, Andrea L. H. Kwa

**Affiliations:** ^1^Department of Pharmacy, Singapore General Hospital, Singapore, Singapore; ^2^Department of Pharmacy, National University of Singapore, Singapore, Singapore; ^3^Saw Swee Hock School of Public Health, National University of Singapore, Singapore, Singapore; ^4^Department of Infectious Diseases, Singapore General Hospital, Singapore, Singapore; ^5^Emerging Infectious Diseases Programme, Duke-National University of Singapore Medical School, Singapore, Singapore; ^6^Singhealth Duke-National University of Singapore Medical School, Medicine Academic Clinical Programme, Singapore, Singapore

**Keywords:** treatment, outcomes, infections, carbapenemase-producing, carbapenem-resistance, *Enterobacterales*

## Abstract

**Background:** Diverse sequence types (ST) and various carbapenemase-producing carbapenem-resistant *Enterobacterales* (CP-CRE) infections, which complicate treatment strategies, have emerged in Singapore. We aim to describe these CP-CRE infections and clinical outcomes according to their carbapenemase types and determine the hierarchy of predictors for mortality that are translatable to clinical practice.

**Methods:** Clinically significant CP-CRE infections were identified in Singapore General Hospital between 2013 and 2016. Retrospectively, all clinically relevant data were retrieved from electronic medical records from the hospital. Univariate analysis was performed. To further explore the relationship between the variables and mortality in different subsets of patients with CP-CRE, we conducted recursive partitioning analysis on all study variables using the “rpart” package in R.

**Results:** One hundred and fifty five patients were included in the study. Among them, 169 unique CP-CRE were isolated. Thirty-day all-cause in-hospital mortality was 35.5% (*n* = 55). There was no difference in the severity of illness, or any clinical outcomes exhibited by patients between the various carbapenemases. Root node began with patients with Acute Physical and Chronic Health Evaluation (APACHEII) score ≥ 15 (*n* = 98; mortality risk = 52.0%) and <15 (*n* = 57; mortality risk = 9.0%). Patients with APACHEII score ≥ 15 are further classified based on presence (*n* = 27; mortality risk = 23.0%) and absence (*n* = 71, mortality risk = 62.0%) of bacterial eradication. Without bacterial eradication, absence (*n* = 54) and presence (*n* = 17) of active source control yielded 70.0 and 35.0% mortality risk, respectively. Without active source control, the mortality risk was higher for the patients with non-receipt of definite combination therapy (*n* = 36, mortality risk = 83.0%) when compared to those who received (*n* = 18, mortality risk = 47.0%). Overall, the classification tree has an area under receiver operating characteristic curve of 0.92, with a sensitivity of 0.87 and specificity of 0.91.

**Conclusion:** Different mortality risks were observed with different treatment strategies. Effective source control and microbial eradication were associated with a lower mortality rate but not active empiric therapy for CP-CRE infection. When source control was impossible, definitive antibiotic combination appeared to be associated with a reduction in mortality.

## Introduction

The growing incidence of carbapenem-resistant Gram-negative bacilli (CRGNB) is an urgent global healthcare challenge today (Paterson and Doi, 2007). Prevalence of carbapenem resistance among GNB in South and Southeast Asian countries, including Singapore, is potentially driven by extensive carbapenem use (Hsu et al., 2017). The number of carbapenem-resistant *Enterobacterales* (CRE) colonization cases reported swelled on the back of ramp-up screening within both public and private hospitals in Singapore (Marimuthu et al., 2017). High counts of carbapenemase-producing (CP) CRE were also recovered from the sewage systems of four main hospitals locally (Koh et al., 2015). Heightened surveillance of these organisms is crucial to their management as CRE is associated with increased mortality and limited treatment options (Molton et al., 2013).

Unlike countries in the United States and Europe, where a predominant *Klebsiella pneumoniae* clonal (ST-258) and resistance type (KPC) is observed, there is greater diversity in Singapore (Teo et al., 2016). This greatly complicates management strategies, including the selection of effective combinations for clinical use. Our local CRE is associated with a variety of resistance mechanisms (e.g., various carbapenemases production in IMPs, KPCs, NDMs, OXA-48, OXA-181, OXA-232, and dual-carbapenemase production with or without porin downregulation) and at least 16 sequence types (ST) (Teo et al., 2016).

To date, there is a lack of consensus on treatment recommendations for CRE infections and data is scanty for patients infected with CP-CRE of varied ST and mechanisms of resistance, especially in the types of carbapenemases production (Molton et al., 2013; Tzouvelekis et al., 2014). Our institution, which is the largest tertiary care hospital in Singapore and an international health hub with a diverse CP-CRE landscape (Teo et al., 2016; Marimuthu et al., 2017), has the highest number of CP-CRE infections.

This retrospective cohort study aims to describe our CP-CRE infections, the various treatment strategies, and the outcomes of CP-CRE infections according to their carbapenemase types and determines the hierarchy of predictors for mortality that are translatable to clinical practice.

## Methods

The retrospective cohort study was conducted in Singapore General Hospital (SGH), a 1,700-bed tertiary care hospital in Singapore. SingHealth Institutional Review Board provided approval with waiver of informed consent (CIRB number: 2014/912/F). Hospitalized adult patients (≥18 years old) with clinical CP-CRE infections from 1st January 2013 to 31st December 2016 were included in the study. These patients were identified from a hospital microbiology database. Criteria for inclusion were as follows: (i) documented CP-CRE culture from clinically relevant sterile sites, with exclusion of positive cultures from the urinary system as it was difficult to retrospectively ascertain if the patient had a true infection; (ii) patient exhibited clinical signs and symptoms of sepsis with systemic inflammatory response from a documented or suspected site of infection, as defined in Society of Critical Care Medicine and European Society of Intensive Care Medicine Surviving Sepsis guidelines (Rhodes et al., 2017); (iii) patient received treatment of CP-CRE infection with at least two consecutive doses of antimicrobial, with the exception of single-dose aminoglycosides. In patients with more than one episode of CP-CRE infection, only the first infection was documented and analyzed.

Data was retrieved from electronic hospital medical records. Data includes demographic information, admission details, past medical history, comorbid conditions (according to Charlson Comorbidity Index, CCI; Charlson et al., 1987), past invasive procedure, prior antibiotic or immunosuppressive therapy, infection characteristics, severity on onset according to Acute Physical and Chronic Health Evaluation (APACHE II) (Knaus et al., 1985) and sepsis-related organ failure assessment (SOFA) (Jones et al., 2009), and treatment regimen. Primary outcome measured was 30-day all-cause in-hospital mortality. Secondary outcomes include time to clinical response, microbiological eradication, and occurrence of reinfection in 1 year.

The following terms were defined prior to data collection and analysis. Onset of infection was determined as the day of sampling of the positive CP-CRE culture. Baseline characteristics of patients and infection details were documented on the day of index culture. Infections were categorized according to the European Center for Disease Prevention and Control guidelines (European Centre for Disease Prevention Control, 2016). Prior hospital exposure was dated for the preceding 1 year, while antibiotic use and surgical operations or invasive procedures at bedside were dated 3 months from index culture. Septic shock was defined as sepsis, a life-threatening organ dysfunction caused by dysregulated host response to infection, with circulatory and metabolic dysfunction (Rhodes et al., 2017). Source control includes any intervention that physically removes the infectious source. Empiric and definitive antimicrobial treatments were defined as regimens given prior to or after susceptibility of index culture was available, respectively. Adequate empirical treatment was defined as receipt of an empirical agent that is active *in vitro* against the pathogen and was administered for at least 48 h. Effective combination therapy was the concurrent use of at least one antimicrobial agent that was still active against the CP-CRE. Clinical improvement was defined as complete or partial response as determined by downtrending inflammatory markers and associated resolution of infective symptoms. Microbiological eradication involved a repeat negative culture of the CP-CRE from the same site after the index culture. Reinfection was defined as clinically significant CP-CRE infections occurring after clinical improvement or microbiological clearance of infection, 7-days after and within 1 year of index culture.

Carbapenem-resistant isolates were identified from the hospital microbiological database. The genus was determined using Vitek 2 ID-GN cards (bioMerieux Inc. Hazelwood, MO). Carbapenem susceptibility was determined using disk diffusion and interpreted according to Clinical and Laboratory Standards Institute (CLSI) guidelines. Polymerase chain reaction was conducted to identify the type of carbapenemase production (Hammoudi et al., 2014). Susceptibility of the following antibiotics were tested: amikacin, aztreonam, cefepime, doripenem, ertapenem, imipenem, levofloxacin, meropenem, piperacillin-tazobactam, polymyxin, tigecycline. Susceptibility of tigecycline and polymyxin was determined using Food and Drug Administration breakpoints (Pillar et al., 2008) and interpreted from CLSI breakpoints against *Enterobacterales* (Lat et al., 2011), respectively. Extensive drug resistance is defined as non-susceptibility to at least one agent in all but two or fewer antimicrobial categories (i.e., bacterial isolates remain susceptible to only one or two antimicrobial categories) (Magiorakos et al., 2012).

### Statistical Analysis

All statistical analyses were performed with SPSS (IBM Corp, Version 20.0) and R (Version 3.6.0). Continuous variables were presented as mean and standard deviation for normal distributed data and as median and interquartile range for non-normal distributed data. Categorical variables were presented as number and percentages. For non-normal distributed data, three-group and two-group comparisons were analyzed via Kruskal–Wallis H test and Kruskal–Wallis test, respectively. Categorical variables were analyzed using Pearson's chi-squared test or Fisher's exact test. To determine the independent factors associated with mortality in CP-CRE patients, lasso regression was first used to identify the factors that best predicted mortality (“glmnet” package in R) (Tibshirani, 1996). Once the final model was identified, traditional multivariable logistic regression was performed, and a final two-tailed *p* < 0.05 was 5% level considered to be statistically significant. To further explore the relationship between the variables and mortality in different subset of patients with CP-CRE, we conducted recursive partitioning analysis on all study variables using the “rpart” package in R (Therneau and Atkinson, 1997). To avoid overfitting, the decision tree was pruned based on the complexity parameter associated with minimal error (i.e., when no additional variables achieve further reductions in node impurity). The sensitivity, specificity, and area under the receiver operating characteristic curve (ROC) was tabulated to assess the performance of the final decision tree.

## Results

### CP-CRE Isolates

**Figure 1:**
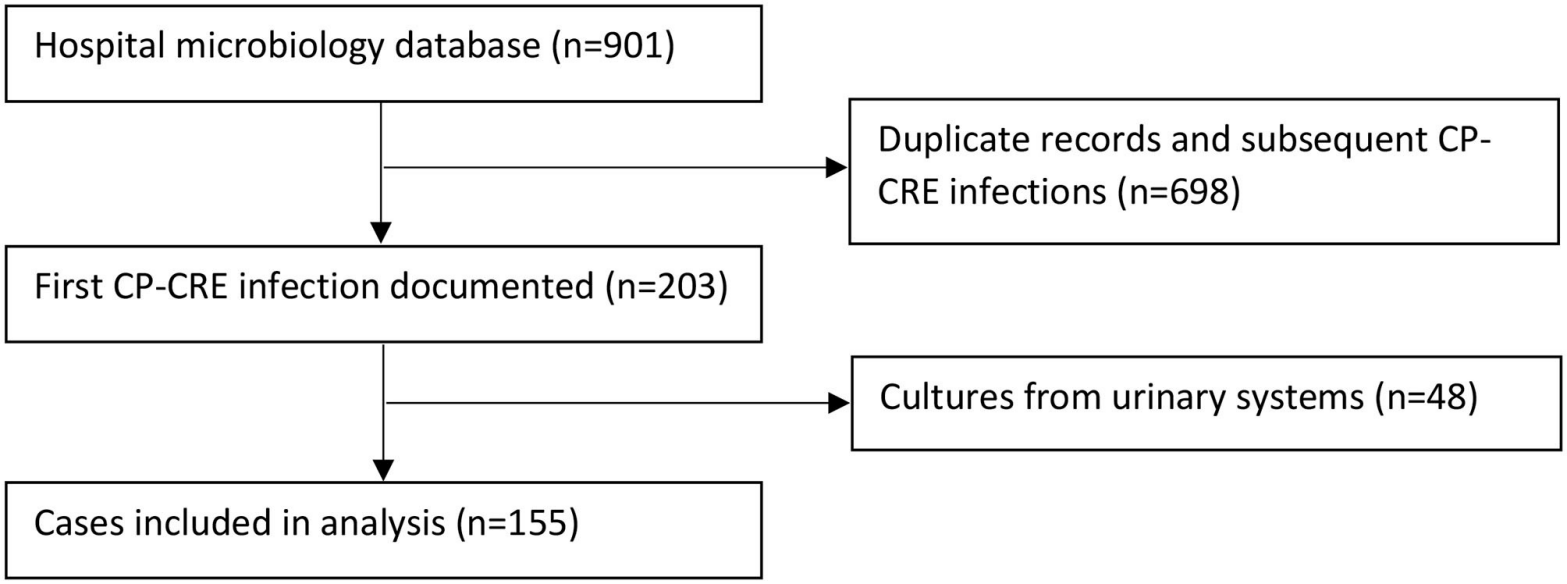
Flowchart of patient selection.

Two hundred and three patients with CP-CRE-positive culture were identified during the study period; 48 did not harbor clinically significant infection. One hundred and fifty-five patients met the inclusion criteria and were included in the study (Figure 1). From these patients, a total of 169 CP-CRE isolates were identified.

### Study Population

The baseline characteristics of the patients and their infections were compared between the various carbapenemases in Table 1. The median age of these patients was 65 years (IQR 56.5–74 years), and the age-adjusted CCI score was 6 (IQR4–8); 43.2% had malignancies. One hundred and thirty-one patients (84.5%) were hospitalized in the preceding 1 year, with 23 (14.8% of the recruited patients) in a foreign hospital. Prior antibiotic exposure was prevalent among 95.5% (*n* = 148) of the patients. Patients, who were infected with KPC-producing isolates, were also more likely to have received surgery compared to patients infected with OXA- and MBL-producing isolates (*p* = 0.035). There was no difference in the severity of illness or any clinical outcomes exhibited by patients between the various carbapenemases. Each eligible patient, whose infection was caused by more than one carbapenem-resistant isolates but with similar carbapenemase production was counted once, under the respective carbapenemase type. However, if any eligible patient had infection involving 2 carbapenemase types, this patient would be included twice, under the respective carbapenemase types for analysis.

**Table 1:** Baseline characteristics of study population and their infections.

	**Total patients**	**Carbapenemase** ***n* = 163**			***P*-value**
	***n* = 155**	^*^KPC ***n* = 86**	^*^MBL ***n* = 49**	^*^OXA ***n* = 28**	
**Demographic**					
Age, median (IQR)	65 (56.5–74)	67 (61–76)	62 (46–69.5)	62.5 (49–71)	0.001
Male (%)	91 (58.7)	48 (55.8)	30 (61.2)	18 (64.3)	0.676
Duration from admission to index culture, median days (IQR)	16 (1–38)	21 (7–40)	12 (1–37)	7 (0.5–26.5)	0.079
**Comorbidities**					
Charlson comorbidity index, median (IQR)	6 (4–8)	6 (5–8)	6 (2–8)	7 (4–8.5)	0.292
Malignancies (%)	67 (43.2)	40 (46.5)	19 (38.8)	11 (39.3)	0.623
Receiving immunosuppressive therapy (%)	29 (18.7)	13 (15.1)	13 (26.5)	5 (17.9)	0.263
**Prior healthcare exposure**					
Hospitalization (%)	131 (84.5)	68 (79.1)	45 (91.8)	26 (92.9)	0.061
Foreign hospitalization (%)	23 (14.8)	3 (3.5)	14 (28.6)	8 (28.6)	<0.001
Antibiotic use (%)	148 (95.5)	84 (97.7)	44 (89.8)	28 (100.0)	0.091
Surgery (%)	65 (41.9)	44 (51.2)	15 (30.6)	9 (32.1)	0.035
Invasive procedure at bedside (%)	138 (89.0)	81 (94.2)	42 (85.7)	22 (78.6)	0.050
**Severity of infection**					
SOFA score, median (IQR)	5 (2–8)	4 (2–8)	5 (2–9)	5 (3.5–7)	0.822
APACHE II score, median (IQR)	17 (12–22.5)	17 (12–25)	15 (12–21.5)	18 (14–20.5)	0.714
Septic shock on infection onset (%)	32 (20.6)	19 (22.1)	10 (20.4)	4 (14.3)	0.671
ICU admission (%)	58 (37.4)	31 (36.0)	19 (38.8)	10 (35.7)	0.943
**Outcomes**					
Length of hospital stay, median (IQR)	48.5 (21.5–74.5)	53 (24–75)	43 (20.5–78)	39.5 (11.5–66)	0.179
Receipt of source control (%)	57 (36.8)	28 (32.6)	22 (44.9)	12 (42.9)	0.309
Clinical response (%)	94 (60.6)	54 (62.8)	30 (61.2)	14 (50.0)	0.478
Time to clinical response, median (IQR)	7 (5–11)	7 (5–10)	9 (3–15)	7 (5–15)	0.529
Microbiological eradication (%)	43 (27.7)	22 (25.6)	14 (28.6)	11 (39.3)	0.380
30-day-all-cause mortality (%)	55 (35.5)	32 (37.2)	13 (26.5)	11 (39.3)	0.379

* *Each eligible patient, whose infection was caused by more than one carbapenem-resistant isolate, but with similar carbapenemase production was counted once, under the respective carbapenemase type. However, if any eligible patient had infection involving 2 carbapenemase types, this patient would be included twice, under the respective carbapenemase types for analysis*.

### Types of CP-CRE Isolates and Their Susceptibilities

The characteristics of CP-CRE isolates are described in Table 2. Among the 174 carbapenemases observed in the 169 isolates, there were 95 (54.6%) KPC, 51 (29.3%) MBL, and 28 (16.1%) OXA carbapenemases. Five out of 169 isolates were CP-CRE carbapenemase co-producers. Four *K. pneumoniae* and 1 *Citrobacter freundii* isolates produced a combination of MBL (specifically NDM) and OXA carbapenemases, each. *K. pneumoniae* was the most commonly encountered *Enterobacterales* (*n* = 86, 50.9%), followed by *Enterobacter* spp. (*n* = 42, 24.9%), *Escherichia coli* (*n* = 32, 18.9%), *Citrobacter* spp. (*n* = 8, 4.7%), and *Serratia marcescens* (*n* = 1, 0.6%). The proportion of extensive drug-resistant KPC-producing isolates (54.7%) was significantly lower (*p* = 0.001) than those of OXA- (82.1%) and MBL- (80.4%) producing isolates. MBL- (OR = 3.39, 95% CI, 1.52–7.55) and OXA-producing (OR = 3.80, 95% CI, 1.33–10.85) isolates demonstrated significantly higher proportion of extensive drug resistance when compared to KPC. The most common infection sites were skin and soft tissue (*n* = 47, 27.8%), followed by intra-abdominal infection (*n* = 43, 25.4%).

**Table 2:** Characteristics of carbapenemase-producing carbapenem-resistant *Enterobacterales* isolates.

	**Total isolates**	**Carbapenemase** ***n* = 174**			***P*-value**
	*n* = 169^*^	**KPC** ***n* = 95**	**MBL** ***n* = 51**	**OXA** ***n* = 28**	
**Species**					
*Klebsiella pneumoniae* (%)	86 (50.9)	49 (51.6)	22 (43.1)	19 (67.9)	0.109
*Enterobacter* spp. (%)	42 (24.9)	29 (30.5)	11 (21.6)	2 (7.1)	0.035
*Escherichia coli* (%)	32 (18.9)	15 (15.8)	11 (21.6)	6 (21.4)	0.624
Others (%)	9 (5.3)	2 (2.1)	7 (13.7)	1 (3.6)	0.013
**Primary site of infection**					
Skin and soft tissue infection (%)	47 (27.8)	22 (23.2)	16 (31.4)	11 (39.3)	0.207
Intra-abdominal infection (%)	43 (25.4)	28 (29.5)	11 (21.6)	6 (21.4)	0.491
Bloodstream (%)	36 (21.3)	18 (18.9)	12 (23.5)	6 (21.4)	0.804
Pneumonia (%)	28 (16.6)	18 (18.9)	7 (13.7)	4 (14.3)	0.674
Others (%)	15 (8.9)	9 (9.5)	5 (9.8)	1 (3.6)	0.662
**Resistance**					
Extensive drug resistance (%)	111 (65.7)	52 (54.7)	41 (80.4)	23 (82.1)	0.001

* *Of 169 isolates, there were 5 co-producers for carbapenemases, with two carbapenemases in each isolate*.

The susceptibilities of the isolates are described in Table 3. The isolates were highly resistant to all carbapenem and cefepime, with 65.7% (*n* = 111) demonstrating extensive drug resistance. Susceptibilities to tigecycline and polymyxin B were only performed in some isolates upon request. As some patients died, or were transferred out of hospital, the susceptibilities of polymyxin B and tigecycline were not requested for. However, among those tested, susceptibilities remained high for tigecycline at 78.1% (100/128 tested) and polymyxin B at 90.6% (115/127 tested). Susceptibilities of MBL-producing isolates remained high toward polymyxin B (88.1%, 37/42 tested) and tigecycline (70.7%, 29/41 tested). The susceptibilities of polymyxin B, tigecycline, and amikacin for KPC-producing isolates remained above 85%, while levofloxacin's susceptibility was notably at 67.9% (38/56 tested). Only 1 KPC-producing isolate demonstrated pan-drug resistance while the 5 carbapenemase co-producers were all extensive drug resistant.

**Table 3:** Antimicrobial susceptibility of carbapenemase-producing carbapenem-resistant *Enterobacterales* isolates.

**Antibiotic**	**Overall susceptibility (%)**	**Carbapenemase (%)**		
		**KPC**	**MBL**	**OXA**
Levofloxacin	46.9	67.9	21.4	12.5
Cefepime	7.7	8.5	2.0	14.3
Ertapenem	1.2	1.1	0.0	3.6
Imipenem	3.1	1.4	0.0	15.0
Meropenem	6.1	7.5	0.0	12.0
Doripenem	5.0	3.6	3.2	12.5
Tigecycline	78.1	86.8	70.7	62.5
Polymyxin B	90.6	91.0	88.1	95.7
Amikacin	74.5	92.5	55.6	40.0
Piperacillin-tazobactam	1.9	1.6	3.7	0.0
Aztreonam	5.4	0.0	14.8	11.1

### Treatment Regimens

One hundred and fifty-two (98.1%) patients received empiric antibiotic therapy, of which 106 (68.4%) had monotherapy. Of the 3 patients not empirically treated, 1 demised within 24 h of index culture, another was terminally discharged, while the last patient was initiated on antibiotics only after culture results were released. When culture and susceptibilities results were back, only 43 (27.7%) patients were found to be receiving adequate empirical therapy, and out of which, 18 (41.9%) and 25 (58.1%) patients received adequate empirical monotherapy and combination therapy, respectively.

Definitive treatment regimens are described in Table 4. Seventy-one percent of patients (*n* = 110) received definitive treatment based on culture results. Definitive monotherapy and combination therapy were administered to 45 (29.0%) and 65 (41.9%) patients, respectively. Tigecycline and polymyxin-containing combination therapy were equally common in the treatment of skin and soft tissue infections while polymyxin-containing combination therapy were most frequently administered for intra-abdominal infections. Only 4 and 9 patients received polymyxin and tigecycline monotherapy, respectively. Among these 13 patients, 6 underwent source control interventions, while the rest (7 patients) demised within 30 days of index culture. There were 3 patients who were treated with meropenem as their CP-CRE exhibited ertapenem resistance but remained susceptible or intermediately susceptible to meropenem. Out of these 3 patients, only 1 patient had surgical debridement and survived, while the other 2 died at 14- and 15-days of infection, respectively. Out of the 15 patients who were treated appropriately with culture-directed monotherapy with fluoroquinolones, 4 patients demised. Only 1 patient, out of these 4 demised, had active surgical source control, while 9 out of the 11 patients who survived had active source control.

**Table 4:** Definitive antibiotic regimen prescribed for patients in this study.

**Antibiotic regimens**	**Overall (%)** ***n* = 155**
**Monotherapy**	45 (29.0)
Fluoroquinolone	15 (9.7)
Aminoglycoside	13 (8.4)
Tigecycline	9 (5.8)
Polymyxin B	4 (2.6)
Carbapenem	3 (1.9)
Cefepime	1 (0.6)
**Combination therapy**	65 (41.9)
Polymyxin B containing	48 (31.0)
Polymyxin B + carbapenem	30 (19.4)
Polymyxin B + tigecycline	6 (3.9)
Polymyxin B + carbapenem + aminoglycoside/fluoroquinolone	4 (2.6)
Polymyxin B + tigecycline + aminoglycoside/fluoroquinolone	4 (2.6)
Polymyxin B + tigecycline + carbapenem	2 (1.3)
Polymyxin B + aminoglycoside/fluoroquinolone	2 (1.3)
Non-polymyxin B containing	17 (11.0)
Tigecycline + aminoglycoside/fluoroquinolone	6 (2.6)
Carbapenem + aminoglycoside/fluoroquinolone	3 (1.9)
Tigecycline + carbapenem	3 (1.9)
Aminoglycoside + fluoroquinolone	3 (1.9)
Cephalosporin + aminoglycoside	2 (1.3)
**No definitive antibiotic therapy**	45 (29.0)

Forty-five (29.0%) patients did not receive definitive antibiotic therapy. Of which, 11 (24.4%) patients underwent source control interventions and survived; 6 (13.3%) patients, with CP-CRE as part of their polymicrobial cultures, were continued with their empiric non-active antibiotic therapy as they had demonstrated clinical improvement by the time their microbiological cultures and their susceptibilities were known. The remaining 28 (62.2%) were patients who had demised prior to culture results being made available, patients who were transferred to another hospital, or patients on palliative care who were managed expectantly.

### Outcomes

During the onset of infection, 58 (37.4%) required ICU admission with a median APACHE II score of 17 (IQR 12–22.5), and 32 (20.6%) presented with septic shock. The overall 30-day all-cause in-hospital mortality rate was 35.5% (*n* = 55). Of these 55 cases, 24 patients succumbed to the infection. The overall clinical response rate was 60.6% (*n* = 94), after a median duration of 7-days (IQR 5–11-days) from infection onset (when index culture was sent). Microbiological clearance was achieved in 43 patients (27.7%) while reinfection with CP-CRE occurred in 15 (9.7%) patients. There were no significant differences in 30-day all-cause mortality rates among the patients who were infected with different carbapenemases producing *Enterobacterales*. From Table 5, 30-day all-cause in-hospital mortality was significantly associated with older patients, ICU admission, septic shock, higher CCI score, APACHE II score or SOFA score, pneumonia infection, patients not receiving definitive combination therapy and those without clinical response, microbiological clearance, or source control interventions.

**Table 5:** Comparison of characteristics between survivors and non-survivors.

	**Survivor** ***n* = 100**	**Non-survivor** ***n* = 55**	***P*-value**
**Demographics**			
Age, median (IQR)	63 (53.5–74)	68 (62–77)	0.003
Male (%)	63 (63.0)	28 (50.9)	0.144
**Comorbidities**			
Charlson comorbidity index, median (IQR)	5 (4–8)	7 (6–9)	0.001
Malignancies (%)	46 (46.0)	21 (38.2)	0.347
Receiving immunosuppressive therapy (%)	19 (19.0)	10 (18.2)	0.901
**Prior healthcare exposure**			
Hospitalization (%)	86 (86.0)	45 (81.8)	0.491
Foreign hospitalization (%)	16 (16.0)	7 (12.7)	0.583
Antibiotic use (%)	96 (96.0)	52 (94.5)	0.699
Surgery (%)	47 (47.0)	18 (32.7)	0.085
Invasive procedure at bedside (%)	89 (89.0)	49 (89.1)	0.986
**Primary site of infection**			
Bloodstream	17 (17.0)	16 (29.1)	0.079
Pneumonia	12 (12.0)	15 (27.3)	0.016
Skin and soft tissue	31 (31.0)	12 (21.8)	0.222
Intra-abdominal	29 (29.0)	10 (18.2)	0.138
Others	11 (11.0)	2 (3.6)	0.114
**Severity of infection**			
SOFA score, median (IQR)	4 (1–5)	8 (5–13)	<0.001
APACHE II score, median (IQR)	14 (10–18)	28 (17–33)	<0.001
Septic shock on infection onset (%)	8 (8.0)	24 (43.6)	<0.001
ICU admission (%)	28 (28.0)	30 (54.5)	0.001
**Infection characteristics**			
Carbapenemase			
KPC (%)	54 (50.5)	32 (57.2)	0.418
MBL (%)	36 (33.6)	13 (23.2)	0.168
OXA (%)	17 (15.9)	11 (19.6)	0.546
Extensive drug resistance (%)	65 (65.0)	36 (65.5)	0.955
**Treatment received**			
Active empiric therapy (%)	23 (23.0)	20 (36.4)	0.075
Definitive monotherapy (%)	30 (30.0)	15 (27.3)	0.720
Definitive combination therapy (%)	49 (49.0)	16 (29.1)	0.016
Source control (%)	49 (49.0)	8 (14.5)	<0.001
**Outcomes**			
Length of hospital stay, median (IQR)	57.5 (34–87)	28 (11–59)	<0.001
Clinical response (%)	88 (88.0)	6 (10.9)	<0.001
Time to clinical response, median (IQR)	7 (5–11)	9 (7–11)	0.675
Microbiological eradication (%)	36 (36.0)	7 (12.7)	0.002

### Risk Factors for Mortality

**Figure 2:**
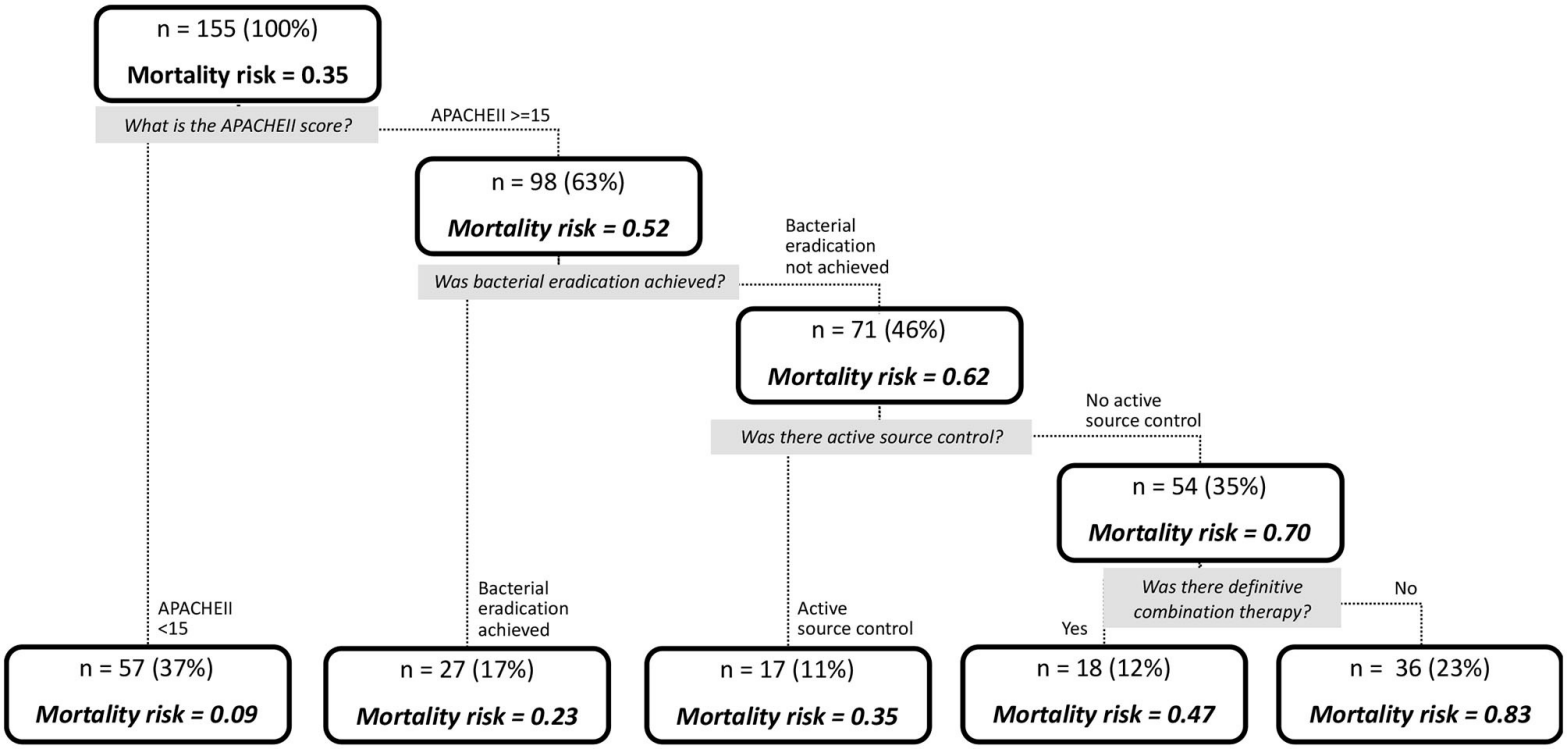
Tree diagram depicting the likelihood of mortality in patients with CP-CRE infections, determined by recursive partitioning.

The results of the multivariable analysis are shown in Table 6. As shown, the presence of source control (OR, 0.258; 95% CI, 0.093–0.661), presence of microbial eradication (OR, 0.176, 95% CI, 0.053–0.504), and receipt of definitive combination therapy (OR, 0.391, 95% CI, 0.162–0.906) were associated with a significant lower risk for 30-day all-cause in-hospital mortality, while APACHE II score ≥15 (OR, 8.755, 95% CI, 3.573–31.997) was a significant predictor for mortality. Using binary recursive partitioning, the final classification tree for mortality in CP-CRE patients included 4 study variables and is shown in Figure 2. The root node classified the patients with APACHEII score ≥15 (*n* = 98; mortality risk = 52.0%) and <15 (*n* = 57; mortality risk = 9.0%). Patients with APACHEII score ≥15 are further classified based on presence (*n* = 27; mortality risk = 23.0%) and absence (*n* = 71, mortality risk = 62.0%) of bacterial eradication; in the subset of patients without bacterial eradication, patients were further subdivided based on presence (*n* = 17, mortality risk = 35.0%) and absence (*n* = 54, mortality risk = 70.0%) of active source control. In patients who did not achieve active source control, the risk of mortality was higher for the patients that did not receive definite combination therapy (*n* = 36, mortality risk = 83.0%) when compared to those who received definite combination therapy (*n* = 18, mortality risk = 47.0%). Overall, the classification tree has an area under ROC of 0.92, with a sensitivity of 0.87 and specificity of 0.91.

**Table 6:** Multivariable logistic regression of predictors for 30-day all-cause mortality in CP-CRE infections.

**Variable**	**Odds ratio (95% confidence interval)**	***P*-value**
Presence of active source control	0.258 (0.093–0.661)	0.022
APACHEII score ≥ 15	8.755 (3.573–31.997)	0.006
Presence of microbial eradication	0.176 (0.053–0.504)	<0.001
Use of definitive combination therapy	0.391 (0.162–0.906)	0.031
Presence of bloodstream infection	2.295 (0.762–7.693)	0.154

## Discussion

### Heterogeneous CP-CRE Infections in Singapore

Unlike countries with a predominant carbapenemase reported (van Duin and Doi, 2016), Singapore, an international health hub, has to deal with a more diverse range of CP-CREs at our healthcare institutions (Molton et al., 2013; Marimuthu et al., 2017). There are 3 predominant carbapenemases (KPC, MBL, OXA-producing) observed in the isolates from our center. Majority of our patients with CRE infections were infected with KPC-producing strains (54.6%) (Hsien Koh et al., 2013), and this was usually associated with prior surgical procedure. However, there was also a significant proportion of MBL isolates (29.3%). Approximately one-sixth of the study population had prior foreign hospital exposure which was significantly associated with MBL infections, and half of these patients were from India or Bangladesh where MBL carbapenemase predominates (Lascols et al., 2011; Snyder et al., 2016; Islam et al., 2017). Among the patients with OXA isolates, ~64.3 and 28.6% had prior local and foreign hospitalization, respectively. Surgery compromises the protective barriers and has been well-established to be associated with infections by CP-CRE (Di Carlo et al., 2013; da Silva et al., 2016; Hilliquin et al., 2018). Locally, we have observed that patients infected with KPC isolates were also more likely to have received prior surgical operations, when compared to the OXA or MBL isolates.

Given the heterogeneity of CP-CRE infections and its associated morbidities and mortality, we applied recursive partitioning to identify predictors of outcomes in CP-CRE infections in a simple and intuitive manner. Translating this to clinical practice, we found that in severely ill (i.e., APACHEII score ≥ 15) patients who could not achieve bacterial eradication and did not receive active source control, the use of definitive antibiotic combinations appeared to improve clinical outcomes with a reduction in mortality. In an environment of diverse mechanisms of resistance, individualized and target therapy for CP-CRE infections, guided by antimicrobial combination testing, is the way forward.

### Degree of Resistance

Depending on the type of carbapenemase production, the antimicrobial susceptibility profile of the various CP-CRE isolates may differ. MBL-producing CP-CRE isolates were at more than 3 times more likely to be extensively drug resistant compared to KPC-producing isolates, likely attributed by the wider array of resistant genes present compared to other carbapenemases (Nordmann et al., 2011; Tzouvelekis et al., 2014). Similarly, 82.1% OXA-producing CP-CRE was extensive-drug resistant; this was at more than 3 times than KPC-producing isolates. This could be of clinical significance, given the mortality associated with OXA-48 producing CP-CRE pan-drug resistance infections (Stewart et al., 2018; Sah et al., 2019). The treatment armamentarium shrinks considerably, and the medical team is left with polymyxin, tigecycline, and to a lesser extent aminoglycoside as our only treatment options.

Interestingly in our study, more than 67% of our KPC-producing isolates retained susceptibility to levofloxacin, which is surprising considering that fluoroquinolone resistance globally is frequently mediated by prevalent plasmid or chromosomal mutations (Endimiani et al., 2008; Morrill et al., 2015; Muggeo et al., 2018). Having said this, there are also reports of fluoroquinolone activity against KPC-producing *Enterobacterales* and they provide an additional treatment option (Vasoo et al., 2015).

### Antibiotic Treatment

In our cohort, approximately only one-quarter of the cohort received adequate empirical treatment, which is comparatively lower than other CP-CRE studies (Nordmann et al., 2011; Tzouvelekis et al., 2014). One of the possible reasons is that although CP-CRE infections have increased in prevalence over the years (Teo et al., 2016), CP-CRE infections are still uncommon due to heightened infection control measures and ongoing surveillance nationwide; the rates of CP-CRE infection stand at 7% (Cai et al., 2017). By far, infections with extended spectrum beta-lactamase-producing pathogens are more common at approximately 38% (Cai et al., 2017). Polymyxin and tigecycline are not used upfront as our first line antibiotics in managing severe infections.

### Treatment Regimens

In general, culture-directed monotherapy with polymyxin or tigecycline was not frequently practiced at our center, despite their susceptibilities toward isolates. This practice is driven by reports of higher treatment failure rates and increased risk of further resistance with monotherapy when compared to combination therapy (Sun et al., 2013; Tumbarello et al., 2015). Similarly, mortality with monotherapy appears to be high. In addition, we had observed that 7 out of 13 patients, with appropriate monotherapy (with polymyxin or tigecycline) administered and no source control interventions, had demised. Surgical removable of infection source where possible should be the primary treatment.

The notable use of either intravenous or oral formulations of levofloxacin and ciprofloxacin as monotherapy or part of the combination treatment in our institution which was based on the high rate of fluoroquinolone susceptibility was observed, especially among our KPC-producing isolates. This is in contrast to observations elsewhere that CP-CRE isolates commonly exhibit resistance to fluoroquinolones (Morrill et al., 2015).

Regardless of the type of infection, the most common combination utilized was carbapenem paired with polymyxins, supported by data, which supports its use to reduce mortality and increase treatment success (Falagas et al., 2011; Tumbarello et al., 2012). The synergistic effect of polymyxins and carbapenem has been demonstrated *in vitro* (Qureshi et al., 2012). We have also found that definitive combination therapy was associated with a lower 30-day all-cause in-hospital mortality rate if active source control was not possible, similar to previous observation elsewhere (Lee and Burgess, 2012). This highlights the importance of individualized and targeted therapy for CP-CRE infections and the need for combination testing, especially in healthcare institutions who are treating a diverse range of CP-CRE infections. Of note, use of definitive carbapenem monotherapy to treat carbapenemase-producing *Enterobacterales*, which remains carbapenem-susceptible during testing, is concerning. Tzouvelekis et al. reported that using carbapenem monotherapy to treat such *Enterobacterales* (especially OXA-48 producing isolates) are often associated with treatment failures (Tzouvelekis et al., 2014).

### Source Control

Source control is vital for the treatment of CP-CRE infections. It was found to be significantly associated with reducing 30-day mortality and clinical improvement in approximately one-quarter of patients not receiving definitive antibiotics therapy (*n* = 11, 24.4%). Higher rates of source control (39.3% vs. 30.2%) among patients on inadequate empiric therapy might have contributed to better clinical rates of improvement (66.1% vs. 46.5%) when compared to patients on adequate empiric treatment. This seems to further reiterate that source control ought to be primary modality of treatment if possible. The inability to perform any form of source control for patients with pneumonia could have contributed to a higher 30-day all-cause mortality (55.6%, *p* = 0.016) compared to other types of infection studied.

### 30-Day All-Cause Mortality

The 30-day all-cause mortality rate of 35.5% within our study was comparable to previous studies on CRE infections (Falagas et al., 2014; Martin et al., 2018). The predictors found to be independently associated with 30-day all-cause mortality include presence of source control, microbial eradication, definitive combination therapy, and APACHE II score, which are consistent with findings from relevant studies (Morrill et al., 2015; Gutiérrez-Gutiérrez et al., 2016). Interestingly, neither presence of extensive drug-resistant phenotype or type of carbapenemases was found to be associated with higher mortality. Using recursive partitioning, we were able to further determine the hierarchy of predictors in a simple and intuitive manner that is easily translatable to clinical practice; most notably, we found that in severely ill (i.e., APACHEII score ≥ 15) patients who could not achieve bacterial eradication and did not receive active source control, use of definitive antibiotic combinations appeared to be associated with a reduction in mortality.

In medical decision-making (classification, diagnosing, etc.), there are many heterogeneous clinical situations, where decision must be made effectively and reliably. Conceptual simple decision-making models with the possibility of automatic learning are the most appropriate for performing such tasks. Decision trees are a reliable and effective decision-making technique that provides high classification accuracy with a simple representation of gathered knowledge, and they have been used in different areas of medical decision-making.

### Limitation

Our study has certain limitations that must be acknowledged. Firstly, this was a retrospective cohort evaluation of treatment outcomes of diverse CP-CRE infections. Secondly, the relatively small representation, from a single tertiary care institution, large academic hospital, may further limit the applicability of our results. Thirdly, the frequent changes within the antibiotic regimens throughout the duration of treatment make systematic assessment of specific treatment regimen difficult. Lastly, with a significant portion of foreign patients, our study is prone to being lost to follow-up as patients get transferred back to their country.

## Conclusion

Our CP-CRE are diverse, resulting in infections that require individualized antibiotic treatment strategies. Effective source control and microbial eradication were associated with a lower rate of 30-day all-cause mortality but not active empiric therapy for CP-CRE infection. If adequate source control could not be implemented safely, definitive use of antibiotic combinations appeared to be associated with a reduction in mortality.

## Data Availability Statement

All datasets generated for this study are included in the article/supplementary material.

## Ethics Statement

The studies involving human participants were reviewed and approved by SingHealth Institutional Review Board provided approval with waiver of informed consent (CIRB number: 2014/912/F). The ethics committee waived the requirement of written informed consent for participation.

## Author Contributions

AK conceived the idea and helped in editing and data analysis. FL and WLa collected patient related data and wrote the manuscript. YL and WLe compiled, analyzed the data, and wrote the manuscript. JC, JT, and YC are involved in microbiological data collection, data analysis, and writing of manuscript. All authors read, vetted, and approved the manuscript.

## Conflict of Interest

The authors declare that the research was conducted in the absence of any commercial or financial relationships that could be construed as a potential conflict of interest.
